# Protonated Defect-Engineered Carbon Nitride Enables Bio-Interface-Enhanced Photodynamic Antibacterial Activity with Potential Periodontal Application

**DOI:** 10.3390/ma19112191

**Published:** 2026-05-22

**Authors:** Ran Li, Guixin Zhu, Junchi Dong, Boyao Lu, Xing Liang

**Affiliations:** State Key Laboratory of Oral Diseases & National Center for Stomatology, West China School, Sichuan University, Chengdu 610041, Chinaponderdjc@163.com (J.D.)

**Keywords:** graphitic carbon nitride, photodynamic antibacterial therapy, protonation, periodontitis

## Abstract

**Highlights:**

**What are the main findings?**

- Protonated defect-engineered g-C_3_N_4_ enhances photodynamic antibacterial activity.
- Protonation strengthens bacteria–material adhesion via bio-interface regulation.
- Protonated defect-engineered g-C_3_N_4_ shows protective effects against inflammation and bone loss in a mouse experimental periodontitis model.
- AFM and omics analyses reveal adsorption-associated antibacterial mechanisms.

**What are the implications of the main findings?**

- Bio-interface modulation is as important as photocatalytic activity in PDT.
- Combining adsorption and ROS generation improves antibacterial efficiency.
- Provides a bio-interface strategy for PDT with potential periodontal application.

**Abstract:**

Periodontitis is a biofilm-associated inflammatory disease that still requires effective local non-antibiotic antibacterial strategies. In this study, we developed a protonated defect-engineered atomic-layered graphitic carbon nitride nano-system (PVCN) for visible light photodynamic antibacterial therapy. Defect engineering was used to improve visible light absorption and photodynamic activity, while protonation introduced a positively biased surface potential to strengthen bacteria–material interactions and enhance interfacial antibacterial efficacy. Under visible light irradiation, PVCN showed increased ROS production, stronger bacterial adhesion, and rapid killing activity against both *Staphylococcus aureus* and *Escherichia coli*, with bactericidal efficiency above 95%. PVCN also disrupted *S. aureus* biofilms and induced membrane damage, intracellular content leakage, and metabolic suppression. Atomic force microscopy and omics analyses further supported enhanced bacterial adsorption as an important contributor to the improved antibacterial efficacy of PVCN. In vitro assays demonstrated preliminary cytocompatibility and hemocompatibility. In a ligature-induced mouse periodontitis model, PVCN reduced bacterial burden, alleviated inflammation, and attenuated alveolar bone loss. These results support PVCN as a promising photodynamic antibacterial material with preliminary therapeutic potential in experimental periodontitis, and highlight bio-interface regulation as a useful strategy for designing efficient carbon nitride-based photodynamic antibacterial materials.

## 1. Introduction

Periodontitis arises from biofilms that trigger chronic inflammation in periodontal tissues and is widely recognized as a leading contributor to tooth loss [1]. About 10% of the worldwide population is affected by severe periodontitis, highlighting its substantial public health burden [2]. Bacterial infection is the primary driver of disease initiation and progression [3]. Owing to the complex anatomical structure of deep periodontal pockets, traditional mechanical debridement often requires adjunctive antimicrobial therapy [4]. However, drug resistance limits the use of antibiotics [5]. Therefore, non-antibiotic, locally effective, and bacteria-targeting therapeutic strategies are required for periodontitis management.

Photodynamic antibacterial therapy (PDT) has emerged as a promising non-antibiotic strategy for bacterial infection control. Upon illumination, photosensitizing agents produce various reactive oxygen species (ROS), including singlet oxygen, superoxide anions, and hydroxyl radicals, which directly damage the physiological structure of the bacteria [6,7]. One notable strength of PDT is its broad antibacterial coverage, together with limited tendency to promote resistance emergence and strong spatiotemporal controllability [8,9]. Among available photocatalytic materials, graphitic carbon nitride (g-C_3_N_4_) is considered a promising candidate owing to its visible light response, chemical stability, metal-free composition, and favorable biocompatibility [10,11]. Nevertheless, bulk g-C_3_N_4_ still suffers from limited active-site exposure and inefficient charge separation, which restrict its antibacterial efficiency. Reducing the thickness is an effective approach; however, the quantum size effect leads to a reduced visible light response and inadequate use of light energy [12,13]. Rational nanostructure engineering has been widely used to improve catalytic performance in different energy- and environment-related catalytic systems [14]. Defect engineering has therefore been introduced to regulate the band structure and charge-carrier behavior of g-C_3_N_4_ at the atomic level [15,16]. In particular, nitrogen vacancies and cyano groups can compensate for the loss of visible light absorption in nanosheet systems and improve photocatalytic performance [17,18].

However, the short diffusion distance of ROS requires close contact between photosensitizers and pathogens to effectively exert antibacterial functions [19]. Phosphate/carboxylic acid groups negatively charge the bacterial surface [20,21], which repels the negatively charged g-C_3_N_4_ through electrostatic forces, thereby significantly hindering the antibacterial efficacy. Therefore, strengthening the interfacial interactions with bacteria is pivotal for enhancing PDT efficiency. However, existing research remains predominantly focused on optimizing photocatalytic activity, whereas strategies to improve the bio-interface have remained relatively unexplored.

Protonation is an effective regulation strategy that introduces protons into carbon nitride to reconstruct its surface physicochemical properties and has been widely used for performance optimization [22,23]. Proton insertion effectively screens the interlayer π–π interactions, inducing interlayer dissociation and increasing the exposure of active sites [24]. More importantly, protonation markedly alters the surface charge distribution of carbon nitride, converting its intrinsically negative surface into a positively biased state, which is favorable for electrostatic interactions with negatively charged microorganisms, such as bacteria [25]. This pronounced adsorption enhancement shortens the effective diffusion distance of the photo-generated reactive species and provides a critical interfacial foundation for efficient photocatalytic antibacterial processes.

In this study, we developed a protonated defect-engineered atomic-layered g-C_3_N_4_ nano-system (PVCN) for visible light photodynamic antibacterial therapy. Defect engineering was introduced to enhance the visible light photodynamic activity of atomic-layered g-C_3_N_4_, while protonation was used to generate a positively biased surface and strengthen interactions with negatively charged bacteria. Thus, PVCN was designed to improve antibacterial performance through both enhanced photodynamic activity and strengthened bacteria–material interfacial adsorption. We further evaluated its therapeutic efficacy in a ligature-induced periodontitis model and explored bacterial response to the combined PVCN plus irradiation treatment by omics analysis. Collectively, this study aimed to establish a bio-interface-enhanced carbon nitride-based photodynamic antibacterial strategy with potential periodontal application.

## 2. Materials and Methods

### 2.1. Preparation and Characterization of PVCN

**Scheme 1:**
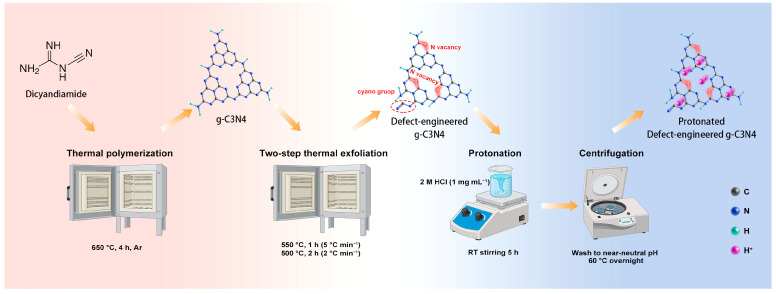
Schematic illustration of the preparation process of PVCN.

The preparation route of PVCN is schematically illustrated in Scheme 1. VCN was prepared by thermal polymerization of dicyandiamide at 650 °C for 4 h under an Ar atmosphere, followed by two-step thermal exfoliation at 550 °C for 1 h (5 °C min^−1^) and 500 °C for 2 h (2 °C min^−1^) to obtain vacancy-rich nanosheets. For protonation, VCN was dispersed in 2 M HCl (1 mg mL^−1^), sonicated for 30 min, stirred at room temperature for 5 h, centrifuged, washed with deionized water to near-neutral pH, and dried at 60 °C overnight to yield PVCN.

Morphology and microstructure were examined by scanning electron microscopy (SEM, JSM-6510LV, JEOL, Tokyo, Japan) and transmission electron microscopy (TEM, FEI Talos, Hillsboro, OR, USA). Crystal structure was analyzed by X-ray diffraction (XRD, D8A25, Bruker, Karlsruhe, Germany), surface charge by zeta potential analysis (Nano ZS90, Malvern, Malvern, UK), functional groups by Fourier-transform infrared spectroscopy (FTIR, Nicolet 6700, Thermo Fisher, Waltham, MA, USA), surface chemical states by X-ray photoelectron spectroscopy (XPS, AXIS Ultra DLD, Kratos, Manchester, UK), and optical properties by UV–vis DRS (Shimadzu UV3600, Kyoto, Japan).

Photodynamic activity was evaluated by electron spin resonance (ESR, EMX-Plus, Bruker) and rhodamine B (RhB) degradation. The generation of ·OH and ·O_2_^−^ was detected under dark and 30 min of visible light irradiation. For RhB degradation, 10 mg of catalyst was added to 100 mL of RhB solution (10 mg L^−1^). After 10 min of dark adsorption, the suspension was irradiated with a xenon lamp (420–780 nm, 300 mW cm^−2^) for 60 min. At designated intervals, 5 mL aliquots were centrifuged at 10,000 g for 10 min, and the absorbance of the supernatant was measured by UV–vis spectrophotometry (UV2800).

### 2.2. In Vitro Antibacterial Evaluation

*Staphylococcus aureus* (*S. aureus*, ATCC 25923) and *Escherichia coli* (*E. coli*, ATCC 25922) were grown in Luria–Bertani (LB) medium at 37 °C and adjusted to 1 × 10^9^ colony-forming unit (CFU) mL^−1^.

For planktonic antibacterial assays, bacterial suspensions were incubated with nanomaterials at different concentrations in 24-well plates, with or without visible light irradiation (300 mW·cm^−2^). At 5, 10, and 15 min, 100 μL aliquots were serially diluted with phosphate-buffered saline (PBS), plated on agar, and cultured at 37 °C for 24 h. Relative bacterial viability was calculated as:

Relative bacterial viability (%) = (CFU_sample/CFU_control) × 100%(1)

CFU_sample and CFU_control represent the colony counts of the treated groups and the control group (bacteria without nanomaterial treatment or irradiation at time 0), respectively. For bacterial growth curves, suspensions treated with 200 µg mL^−1^ nanomaterials for 15 min were further cultured at 37 °C for 18 h, and the optical density at 600 nm (OD_600_) was recorded hourly. All experiments were performed in triplicate.

For morphology observation, bacteria treated with 200 µg mL^−1^ nanomaterials for 15 min were examined by SEM and TEM. SEM samples were fixed in 2.5% glutaraldehyde, dehydrated through graded ethanol, dried, and gold-coated. TEM samples were prefixed in 2.5% glutaraldehyde for 4 h, postfixed in 1% osmium tetroxide for 2 h, dehydrated in acetone, embedded in Epox812, sectioned with diamond knife, and stained with uranyl acetate and lead citrate. The control group consists of untreated bacteria.

Intracellular ATP and extracellular protein leakage were measured using an Enhanced ATP Assay Kit and an Enhanced BCA Protein Assay Kit (Beyotime, Shanghai, China). After treatment with 200 µg mL^−1^ nanomaterials for 15 min, bacteria were centrifuged at 10,000× *g* for 5 min at 4 °C; pellets were used for ATP measurement and supernatants for protein leakage analysis. The control group consists of untreated bacteria, and data without exposure to light after adding materials is also collected.

Anti-biofilm assays were performed using *S. aureus* biofilms formed in 24-well plates after incubation in LB medium at 37 °C for 24 h. Mature biofilms were treated with 200 µg mL^−1^ nanomaterials for 15 min and evaluated by SYTO 9/PI live/dead staining (Thermo Fisher L13152, Waltham, MA, USA) using laser scanning confocal microscopy (LSCM, Fv3000; Olympus, Tokyo, Japan).

Intracellular ROS in bacteria was detected by Reactive Oxygen Species Assay Kit (Beyotime, Shanghai, China). After washing, bacterial suspensions were treated with 200 μg mL^−1^ material under light irradiation for 15 min, and fluorescence intensity was measured using a spectrophotometric microplate reader before and after irradiation.

### 2.3. Bacteria–Material Interaction and Omics Analysis

Tipless atomic force microscopy (AFM) cantilevers (HQ: CSC38/tipless/AlBS, μ-Masch, Estonia) were treated with 0.1% (*w*/*v*) poly-L-lysine for 1 min and then cultured in bacterial suspension for 2 min. Separately, 200 μg mL^−1^ nanomaterial suspension was dropped onto freshly cleaved mica and air-dried. The interaction force measurements were then performed in contact mode using an atomic force microscope (Shimadzu SPM-9700, Kyoto, Japan). For each measurement, 10 randomly selected positions were tested and averaged as one independent value. Each material–bacteria combination was measured using five independently prepared samples.

For omics analysis, *S. aureus* in the treatment group (TR) was exposed to 50 µg mL^−1^ PVCN under visible light irradiation for 10 min, whereas the control group (CL) received no treatment. After washing and centrifugation, bacterial pellets were snap-frozen in liquid nitrogen for 15 min and stored at −80 °C. Total RNA extraction, transcriptome sequencing, metabolite extraction, and subsequent bioinformatics analyses were performed by Personalbio (Nanjing, China).

### 2.4. In Vitro Biocompatibility Assessment

L929 fibroblasts were maintained in Dulbecco’s modified Eagle’s medium (DMEM; Gibco, Waltham, MA, USA), while rat bone marrow–derived mesenchymal stem cells (rBMSCs) were cultured in α-minimum essential medium (α-MEM; Gibco, USA). Both media were supplemented with 10% fetal bovine serum (FBS; Gibco, USA) and 1% penicillin–streptomycin. Cells were incubated at 37 °C in 5% CO_2_.

Cell viability was evaluated by Cell Counting Kit-8 (CCK-8) assay. rBMSCs were cultured with nanomaterials for 1, 2, and 4 days, incubated with CCK-8 reagent for 90 min, and measured at 450 nm. Relative cell viability was calculated as:

Relative cell viability (%) = (OD sample/OD control) × 100%(2)

OD sample and OD control represent the OD_450_ values of the treatment groups and the control group (cells without nanomaterials at 1 day).

For cell morphology, cells were co-cultured with 100 µg mL^−1^ nanomaterials for 1 day, fixed with 4% paraformaldehyde, permeabilized with 0.5% Triton X-100, stained with FITC-Phalloidin (YiSen, Shanghai, China) for 30 min and DAPI (YiSen, Shanghai, China) for 60 s, and imaged by LSCM.

For scratch assay, L929 cells were seeded in 24-well plates at 1 × 10^5^ cells per well and cultured to approximately 95% confluence. A linear wound was created with a sterile 1 mL pipette tip, and fresh medium containing 50 μg mL^−1^ nanoparticles was added. Images were recorded at 0, 18, and 36 h.

For hemolysis assay, whole blood from Sprague–Dawley (SD) rats was diluted to obtain a 2% (*w*/*v*) erythrocyte suspension. Nanomaterials (100 µg mL^−1^) were added to the experimental group, with deionized water and normal saline as positive and negative controls, respectively. After incubation at 37 °C for 4 h, absorbance of the supernatant was measured at 540 nm.

### 2.5. In Vivo Periodontitis Model and Therapeutic Evaluation

Fifteen male C57BL/6 mice (8 weeks old) were obtained from Sichuan University. All animals were housed under specific pathogen-free conditions at 22 ± 2 °C, 50 ± 10% relative humidity, and a 12 h light/dark cycle, with free access to standard chow and water. The mice were acclimated for one week before the experiment. All animal procedures were approved by the Ethics Committee of West China Hospital of Stomatology, Sichuan University (protocol code No. WCHSIRB-D-2025-113; date of approval: 20 February 2025) and were conducted in accordance with relevant institutional guidelines. Experimental periodontitis was induced by placing a 6–0 silk ligature around the cervical region of the maxillary first molar. After two weeks of ligation, mice were randomly assigned to three groups (n = 5 per group). Each mouse received 50 µL nanomaterial suspension (100 µg mL^−1^) or PBS by oral irrigation, followed by visible light irradiation for 15 min every other day for one week.

At the end of treatment, the animals were euthanized, and the maxillae were harvested for bacterial counting, micro-computed tomography (micro-CT; mCT-50, Scanco Medical Inc., Brüttisellen, Switzerland), and histological analysis. The distance from the cemento-enamel junction (CEJ) to the alveolar bone crest (ABC) and bone volume/total volume (BV/TV) were quantified. Blood from randomly selected mice in each group was collected for inflammatory cytokine analysis using an ELISA kit (ZCIBIO, Shanghai, China). Maxillary tissues were decalcified for 4 weeks, embedded in paraffin, and sectioned for hematoxylin and eosin (H&E), tartrate-resistant acid phosphatase (TRAP), and Masson’s trichrome staining.

### 2.6. Statistical Analysis

All data are presented as mean ± standard deviation (SD). Statistical differences among groups were analyzed by one-way analysis of variance (ANOVA) followed by Tukey’s multiple comparison test. A *p* value < 0.05 was considered statistically significant.

## 3. Results

### 3.1. Morphology and Structure Characterization

**Figure 1:**
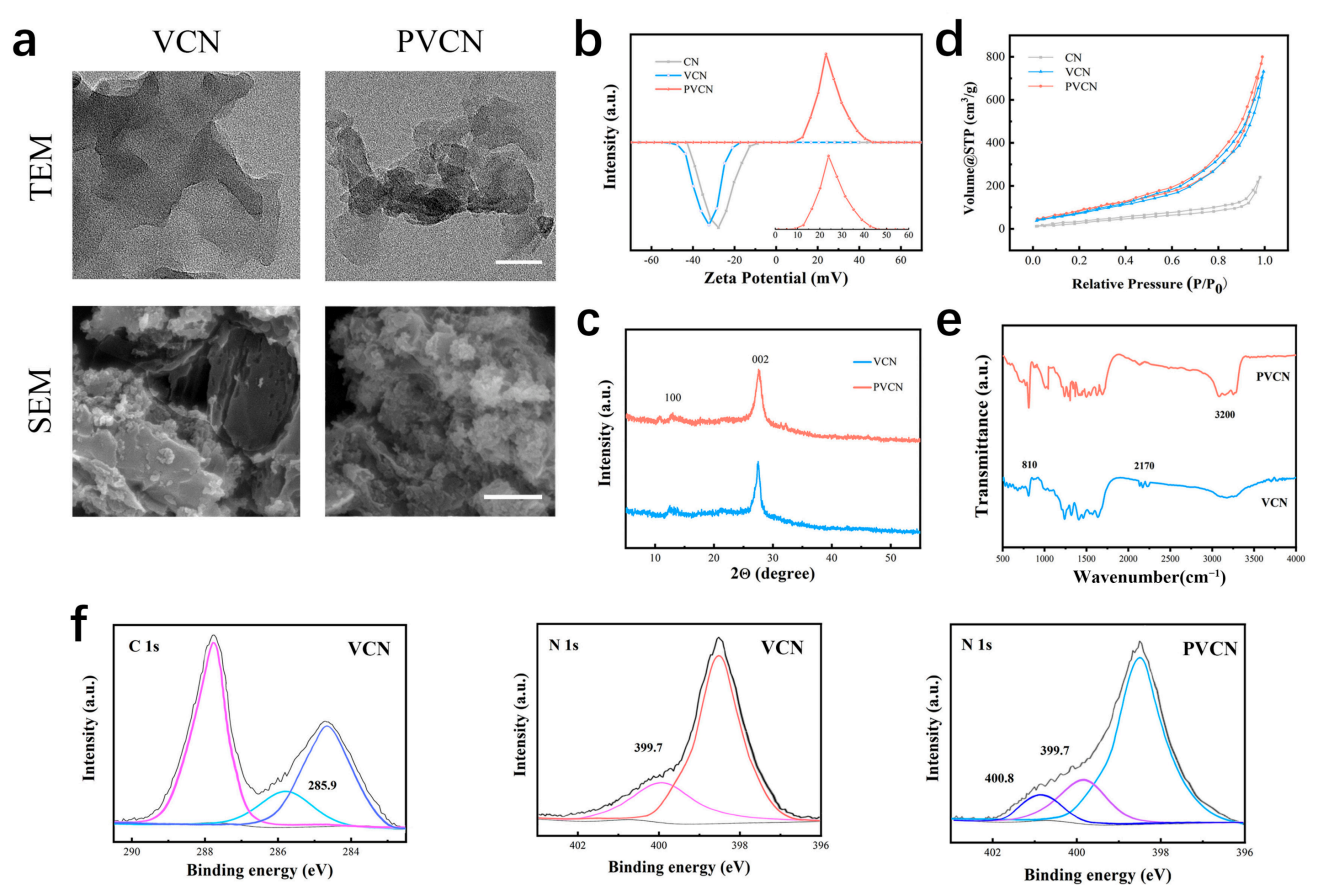
Morphology and characterization of nanomaterials. (**a**) Representative TEM and SEM micrographs of the nanomaterials, with scale bars of 50 nm and 1 μm. (**b**) Zeta potential. (**c**) XRD patterns. (**d**) N2 physisorption isotherms. (**e**) FTIR spectra. (**f**) XPS spectra. From left to right: C 1s in VCN, N 1s in VCN and N 1s in PVCN, the colored lines represent the deconvoluted components corresponding to different chemical states of carbon and nitrogen.

The microstructures of the samples were characterized using TEM and SEM (Figure 1a). The TEM images show that VCN exhibits a typical two-dimensional ultrathin nanosheet morphology with loosely stacked layers, indicating an atomic-level exfoliated structure. In contrast, PVCN retained its overall two-dimensional framework without notable collapse or severe aggregation, whereas the nanosheets displayed notable curling and wrinkling, along with local overlapping and structural reconstruction. SEM observations further revealed that the originally ordered and compact lamellar stacking transformed into a looser and rougher architecture after protonation. The interlayer cohesion was weakened, the edges became fragmented, and the surface evolved into loosely aggregated clusters composed of irregular nanosheets. These changes suggest that protonation enhances interlayer electrostatic repulsion, weakens the original π–π stacking interactions, and promotes nanosheet dispersion and structural rearrangement, rendering the structure more loosely assembled and flexible [26].

Zeta potential measurements were conducted to confirm the effectiveness of protonation (Figure 1b). The surface potential of PVCN shifted from a negative value in VCN to a distinctly positive value, clearly indicating successful proton incorporation and effective modulation of the surface charge characteristics. Following the surface charge analysis, the crystal structure was investigated using X-ray diffraction (XRD). The XRD patterns show that pristine g-C_3_N_4_ (CN) exhibits typical (100) and (002) reflections at 12–13° and 27–28°, corresponding to in-plane tri-s-triazine packing and interlayer π–π stacking (Figure 1c). These peaks were retained for VCN and PVCN, indicating that defect engineering and protonation did not disrupt the layered conjugated framework. However, the (002) peak of PVCN shows reduced intensity and slight broadening, suggesting decreased interlayer ordering due to proton-induced electrostatic repulsion that weakens π-π interactions [27]. Together, these results indicate that the main crystalline framework was preserved, although the interlayer packing became less ordered after protonation. To further examine the structural openness, N_2_ adsorption–desorption isotherms were analyzed (Figure 1d). The N_2_ adsorption–desorption isotherms indicated that VCN had a markedly higher surface area than CN, and PVCN showed a moderate increase in surface area compared with VCN. This trend indicates that defect engineering enhances structural looseness and porosity, and subsequent protonation further weakens interlayer π–π stacking, leading to a more open layered structure [28]. This result is consistent with the reduced (002) peak intensity observed using XRD and SEM.

Subsequently, Fourier-transform infrared spectroscopy (FTIR) and X-ray photoelectron spectroscopy (XPS) were performed to investigate the electronic structure evolution induced by defect construction and subsequent protonation. In the FTIR spectra (Figure 1e), characteristic bands were observed at 810 cm^−1^ and 1200–1700 cm^−1^ in all samples, confirming preservation of the conjugated backbone. The peak at 2170–2200 cm^−1^ in VCN and PVCN corresponded to C≡N stretching, confirming the defect [29]. The enhanced broad band at 3000–3400 cm^−1^ in PVCN indicated successful proton incorporation and strengthened hydrogen bonding. As for XPS (Figure 1f), in the C 1s spectra, the peaks centered at 288.0, 285.9, and 284.7 eV correspond to N=C–N_2_ coordination within the g-C_3_N_4_ framework, C–NH_x_ or cyano groups located at the edges of heptazine units, and C–C bonds, respectively. The component at 285.9 eV becomes more pronounced, suggesting the formation of a defect-related carbon species distinct from N–C=N [30]. The C 1s spectrum of PVCN remained similar to that of VCN, confirming that protonation did not disrupt the carbon framework. In the N 1s region, the peak at ~399.7 eV assigned to N–(C)_3_ species in VCN exhibits a reduced area, reflecting a decrease in tertiary bridging nitrogen. After protonation, PVCN shows a significant enhancement at 400.8 eV along with a further attenuation of the ~399.7 eV component, confirming successful proton incorporation and the formation of positively charged nitrogen species [31]. These results indicate that defects were effectively constructed, protons were successfully introduced, and the crystalline framework was preserved.

### 3.2. Photodynamic Performance and Underlying Mechanism

**Figure 2:**
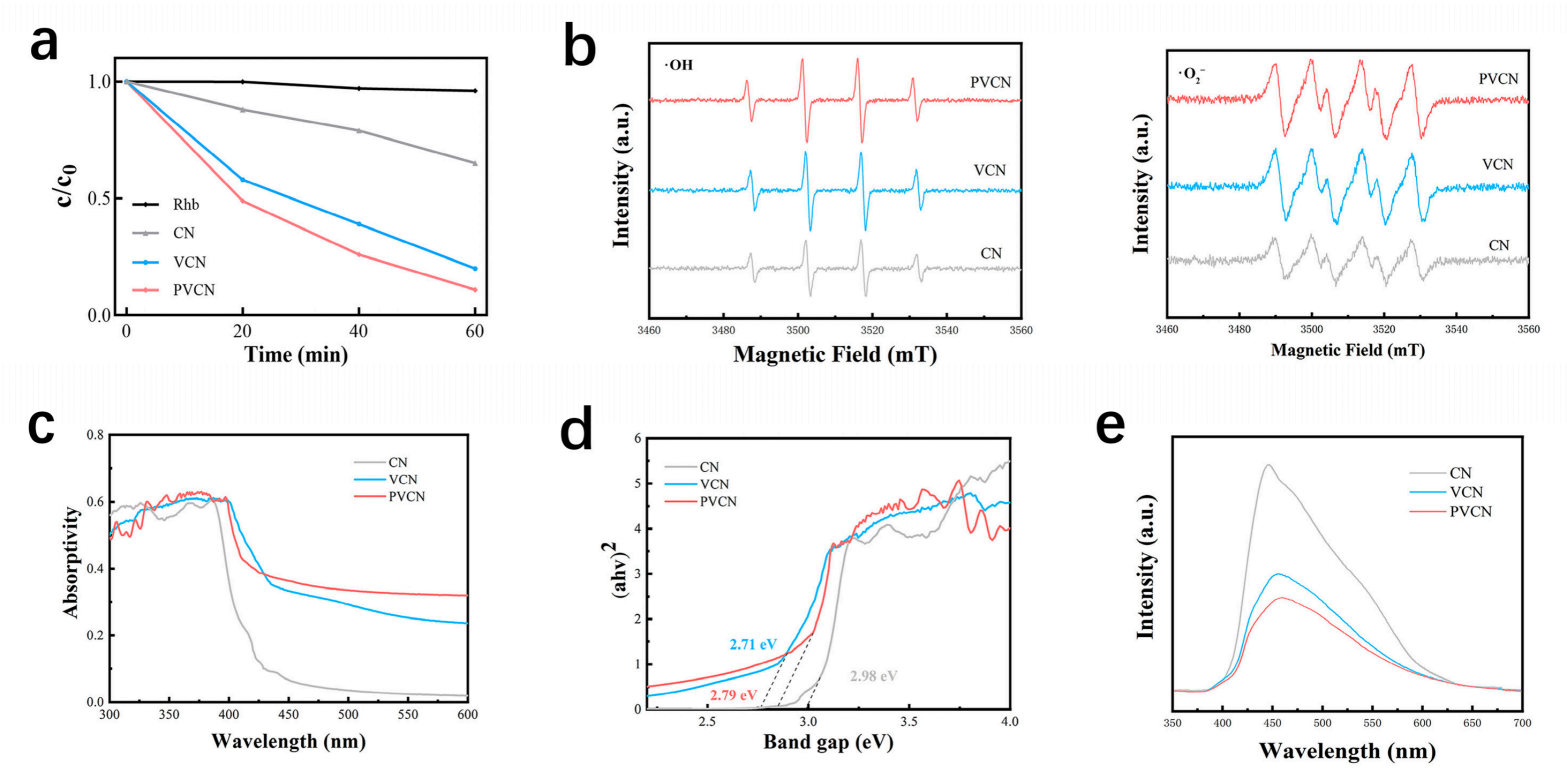
Photodynamic-related characterization of nanomaterials. (**a**) RhB degradation profiles under visible light irradiation in the presence of different nanomaterials, with RhB alone as the control. (**b**) ESR spectra showing ·OH and ·O_2_^−^ generation. (**c**) UV–vis–NIR absorption curves. (**d**) Optical band-gap analysis. (**e**) PL spectra.

The light-induced catalytic behavior of the nanomaterials was systematically evaluated to compare their photodynamic performance. PVCN achieved the highest RhB degradation efficiency under visible light irradiation (Figure 2a), demonstrating its enhanced photocatalytic capability. Moreover, electron spin resonance (ESR) analysis confirmed significantly increased production of ·OH and ·O_2_^−^ radicals in PVCN compared with those in the control groups (Figure 2b). Collectively, these findings verified the superior visible light-driven photodynamic performance of PVCN.

To clarify the origin of this enhancement, UV–vis–NIR diffuse reflectance spectroscopy and photoluminescence (PL) measurements were performed. The UV–vis–NIR results showed that VCN exhibited a distinct red-shift of the absorption edge compared to CN, accompanied by enhanced absorption in the visible region (Figure 2c). The band gap decreased from 2.98 eV for CN to 2.71 eV for VCN, indicating that defect engineering effectively introduces intermediate states and lowers the effective transition energy barrier [32] (Figure 2d). In contrast, PVCN displayed a slightly increased band gap, and its overall absorption intensity was further increased. This enhancement is likely attributable to protonation, which modulates the local electronic density and promotes the electronic transition probability, thereby improving the light-harvesting capability. The PL spectra showed that the emission intensities of both VCN and PVCN were significantly lower than those of CN (Figure 2e). The quenched emission in the VCN indicated that the defect-induced intermediate states promoted charge separation and suppressed radiative recombination [33]. Further reduction in the intensity of PVCN suggests that protonation modulates the electronic distribution and enhances charge transfer, leading to more efficient recombination suppression.

### 3.3. In Vitro Photocatalytic Antibacterial Performance

**Figure 3:**
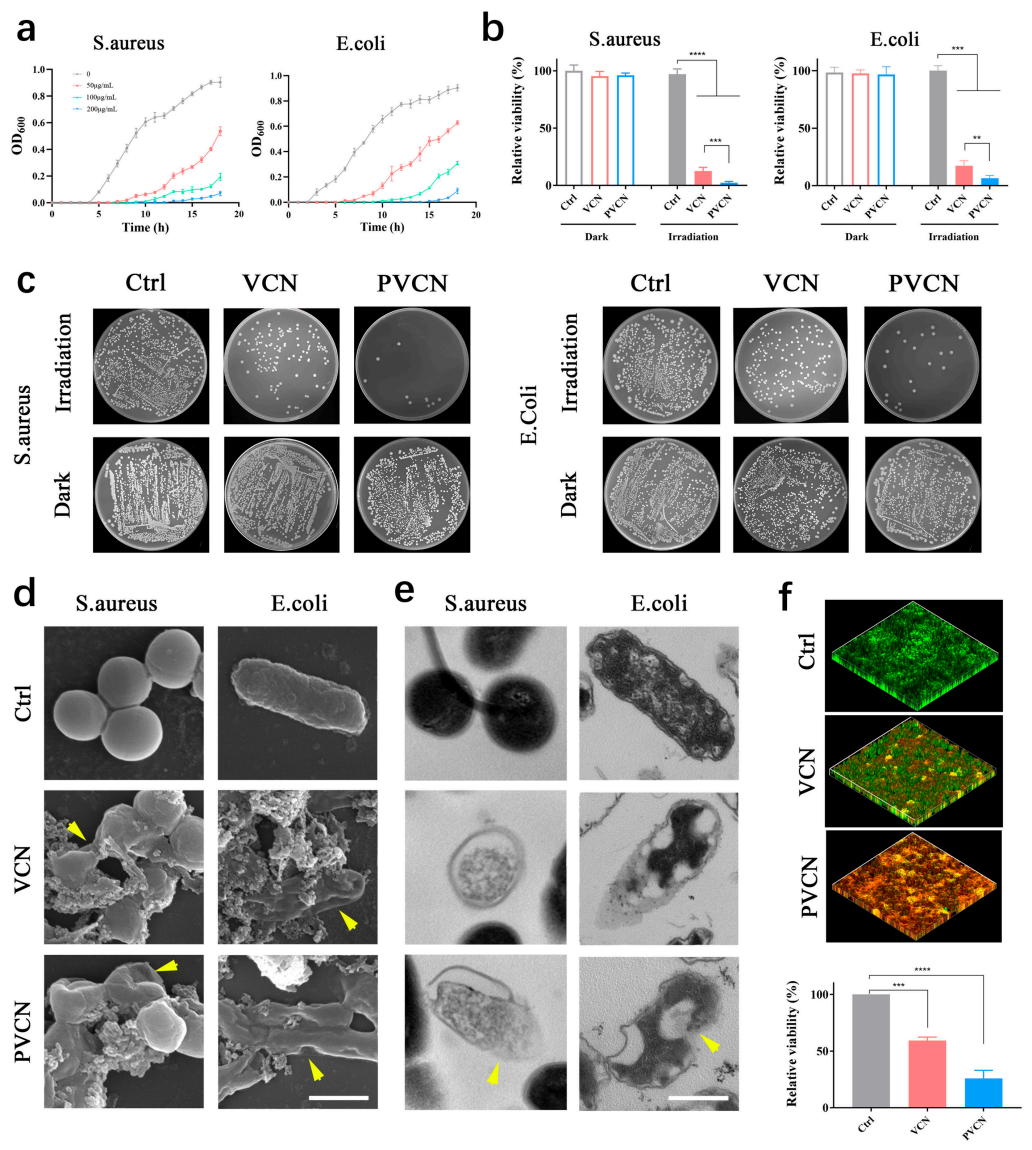
In vitro antibacterial performance of the nanomaterials against *S. aureus* and *E. coli*. (**a**) Growth curves of *S. aureus* and *E. coli* following treatment with different concentrations of nanomaterials. (**b**) Relative viability of bacteria after exposure to the nanomaterials under different conditions. (**c**) Representative colony-forming images of bacteria after treatment with 200 µg mL^−1^ nanomaterials for 15 min. (**d**) SEM images showing the surface morphology of bacteria after treatment with nanomaterials. The yellow arrow indicates surface wrinkling, with scale bars of 1 µm. (**e**) TEM images of bacteria after treatment with nanomaterials. The yellow arrow indicates cellular contents leakage, with scale bars of 300 nm. (**f**) Live/dead staining of *S. aureus* biofilms after treatment with nanomaterials, together with the corresponding quantitative analysis of bacterial viability. Data are presented as mean ± SD (n = 3). ** *p* < 0.01, *** *p* < 0.001, **** *p* < 0.0001.

*Staphylococcus aureus* and *Escherichia coli* were employed as representative Gram-positive and Gram-negative bacteria, respectively, to assess the light-activated antibacterial efficacy of the nanomaterials. First, the bacterial growth curves were monitored by measuring the optical density at 600 nm (OD600) following 15 min of visible light irradiation (Figure 3a and Figure S1A, Supplementary Materials). The results indicated that the nanomaterials markedly inhibited the growth of both bacterial strains. To further quantify antibacterial activity, bacterial survival was determined by colony-forming unit (CFU) counting. There was no significant change in the number of CFU compared with that in the control and dark group. In contrast, the number of bacterial cells decreased in the VCN and PVCN groups. At 15 min, the sterilization efficiency of VCN exceeded 85%, whereas that of PVCN exceeded 95% (Figure 3b,c). This may be attributed to protonation, which modulates the electronic structure, thereby promoting ROS generation, while simultaneously inducing a positive surface potential in PVCN. PVCN maintains good antibacterial activity even with a short exposure time, and its bactericidal activity exceeds 50% after just 5 min. Moreover, the antibacterial effect became progressively stronger with increasing material concentration and longer irradiation time, and the visualized CFU changes are shown in Figure S1B. Additionally, the resistance of *S. aureus* was lower than that of *E. coli*, possibly because Gram-positive bacteria do not have dense outer membranes comprising lipopolysaccharides and lipoproteins [34].

Furthermore, SEM and TEM were used to investigate morphological changes in the bacteria after material treatment (Figure 3d,e). The SEM images showed that the bacteria in the control group exhibited smooth surfaces and intact morphology, whereas the treated bacteria displayed notable surface wrinkling, indicating compromised structural integrity. Notably, the material tightly adhered to bacterial surfaces, enabling direct contact, which was also the basis for the material to exert its PDT effect. TEM further confirmed that after treatment, the bacterial cell membrane/wall underwent deformation or rupture, leading to the cellular contents leakage. Simultaneously, the intracellular structures became disordered, with the appearance of vacuoles and a decrease in density. These results suggest that enhanced bacterial adhesion of the material improves the efficiency of ROS-mediated antibacterial activity.

Antibacterial activity was indirectly evaluated by measuring bacterial adenosine triphosphate (ATP) levels and extracellular protein concentrations [35,36]. As shown in Figure S2a, intracellular ATP levels markedly decreased after treatment, whereas extracellular protein concentrations significantly increased (Figure S2b). These results indicate that the material treatment suppressed normal bacterial metabolic activities and compromised structural integrity, leading to bacterial membrane/wall damage and subsequent intracellular protein leakage, ultimately resulting in bacterial death.

Because biofilm formation is a key feature of periodontal infection, a mono-species *S. aureus* biofilm model was used as a preliminary anti-biofilm assay. Although this model does not reproduce the polymicrobial and anaerobic features of subgingival periodontal biofilms, it provides an initial assessment of the biofilm-disruptive potential of PVCN [37]. Live/dead fluorescence staining results (Figure 3f) showed a pronounced increase in red fluorescence within the treated biofilms, accompanied by reduced integrity and thickness, indicating that the material also exhibited effective disruptive activity against this mono-species biofilm model.

### 3.4. Exploration of Antibacterial Mechanism

**Figure 4:**
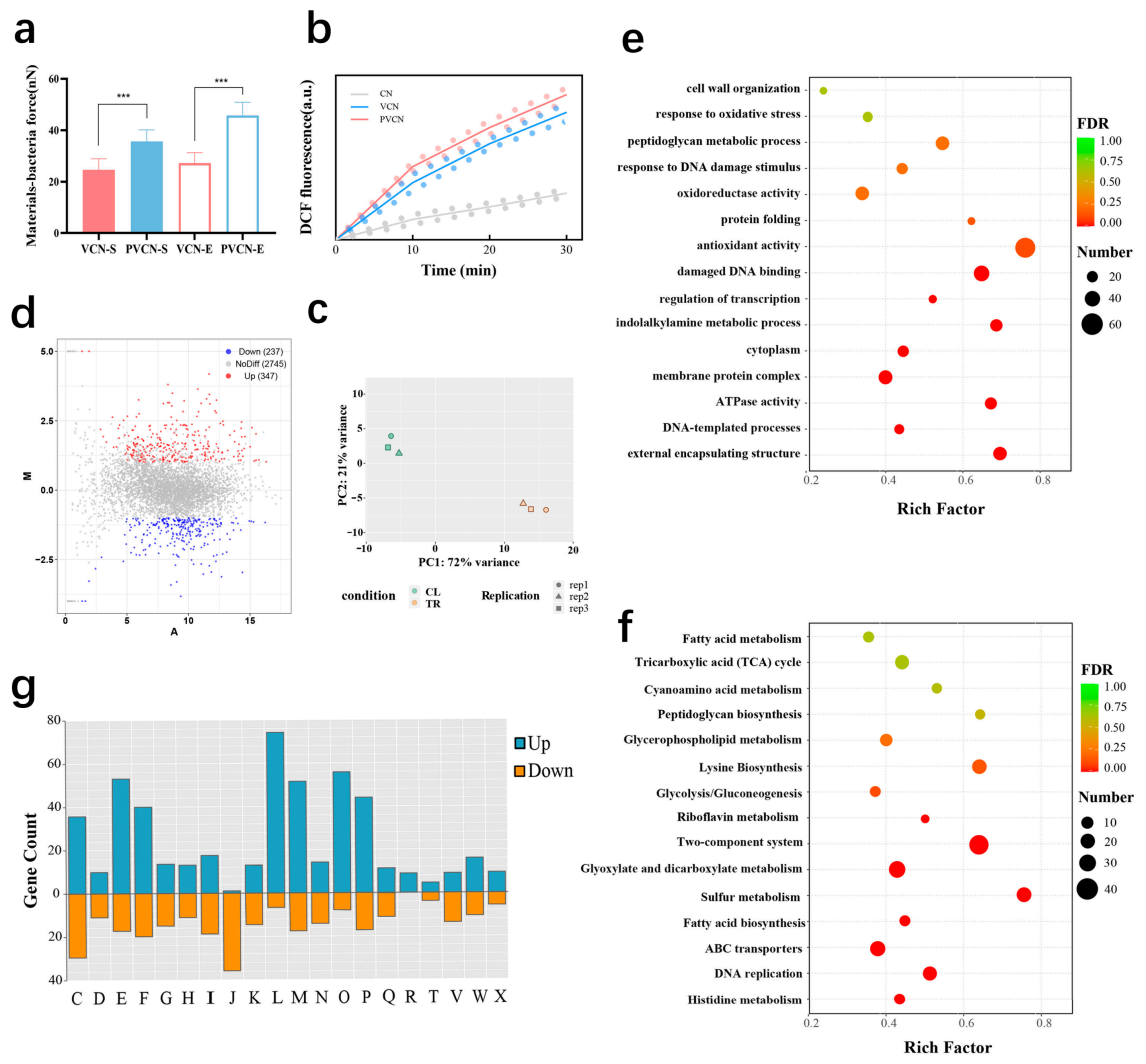
Mechanistic investigation of the PDT-based antibacterial activity of the nanomaterials. (**a**) AFM-based quantification of the interaction forces between nanomaterials and bacterial cells. (**b**) Intracellular ROS levels in bacteria after incubation with nanomaterials under irradiation. (**c**) The PCA plot scores were derived from the transcriptomics of the CL and TR groups. (**d**) MA plot showing the differentially expressed genes (DEGs) between the CL and TR groups. (**e**) GO enrichment analysis of DEGs identified between the CL and TR groups. (**f**) KEGG pathway enrichment analysis of DEGs identified between the CL and TR groups. (**g**) Distribution of DEGs between the CL and TR groups across COG functional categories. Data are presented as mean ± SD (n = 3). *** *p* < 0.001.

To clarify the specific contribution of protonation-mediated bio-interface regulation to the PDT antibacterial process, AFM was employed to quantitatively measure the interaction forces between the material and bacteria [38] (Figure 4a and Figure S3). The adhesion force between PVCN and *S. aureus* exceeded 35 nN, while the interaction force with *E. coli* was approximately 50 nN, both significantly higher than those observed for VCN–bacteria interactions. These results indicate that PVCN exhibited stronger interfacial interaction with bacterial cells. This enhanced interfacial adhesion provides a physical basis for the localized high-density generation of ROS on the bacterial surface, effectively shortening diffusion distance and amplifying oxidative damage [39,40], thereby contributing substantially to the improved antibacterial performance.

Furthermore, intracellular ROS generation was assessed via DCFH-DA fluorescent probe. As shown in Figure 4b, bacterial cells incubated with nanomaterials exhibited stronger fluorescence signals after light exposure. These data indicate that PVCN treatment was associated with stronger intracellular ROS signals after irradiation. Together with the AFM results, these findings suggest that the improved antibacterial efficacy of PVCN is mainly attributable to protonation-enhanced bacteria-material adhesion, which facilitates more efficient local ROS-mediated killing.

To further investigate bacterial responses to the combined PVCN plus irradiation treatment, transcriptomic analysis was performed using *S. aureus*. As illustrated in the principal component analysis (PCA) score plot (Figure 4c), the gene expression signature of the PVCN-treated group (TR) differed from that of the control group (CL). This transcriptomic shift involved 584 differential expression genes, comprising 347 upregulated and 237 downregulated genes, the details of which are summarized in the corresponding MA plot (Figure 4d).

Gene Ontology (GO) enrichment analysis revealed altered biological processes in bacteria following PDT treatment (Figure 4e). Terms related to transmembrane transport, cell membrane and wall organization, oxidative stress response, and DNA damage repair were also significantly enriched. Particularly, the enrichment of terms related to the external encapsulating structure and cell wall organization suggests that PVCN treatment imposed substantial stress on the bacterial envelope. The enrichment of ion transport and other transmembrane transport-associated processes further indicated altered membrane permeability and disruption of ionic homeostasis [41]. These changes are consistent with the intracellular ROS accumulation, SEM/TEM-observed envelope damage in this study, and therefore support the occurrence of ROS-associated oxidative injury and membrane/wall disruption. In contrast, the enrichment of DNA damage repair and some transport-related processes may represent downstream bacterial stress adaptation and repair responses following PDT-induced injury.

The Kyoto Encyclopedia of Genes and Genomes (KEGG) pathway analysis further confirmed that PVCN-mediated PDT caused broad metabolic and envelope-associated perturbations (Figure 4f). Sulfur metabolism and related thiol-associated pathways were significantly enriched, suggesting that sulfur-containing redox systems play a critical role in ROS detoxification. The enrichment of ABC transporters and two-component systems indicated the activation of environmental sensing and regulatory networks, enabling bacteria to modulate membrane stability and ion transport in response to stress [42]. Alterations in fatty acid biosynthesis and metabolism are consistent with membrane damage or remodeling, whereas changes in the tricarboxylic acid cycle and DNA replication suggest metabolic reprogramming and impaired proliferation. These KEGG results were consistent with the GO findings and supported classical ROS-mediated PDT injury, including envelope damage, oxidative stress, and intracellular functional disturbance; however, the enriched metabolic, redox-related, transport, and repair pathways may also reflect downstream adaptive or defensive responses to PDT-induced stress rather than exclusively direct ROS-driven effects.

Clusters of orthologous Groups (COG) classification analysis further confirmed a global functional shift toward a stress-survival mode (Figure 4g). Functional categories associated with cell wall and membrane biogenesis, inorganic ion transport, and DNA replication and repair were significantly enhanced, whereas translation-related functions were suppressed. This pattern indicates a transition from an active proliferation to a defensive state focused on maintaining cellular integrity and survival after combined PVCN plus irradiation treatment.

Since this analysis was designed to characterize the overall bacterial response after effective PVCN-mediated PDT treatment, these transcriptome changes are explained as a stress response of bacteria to PDT-induced oxidative damage, rather than quantifying individual contributions of material adhesion, protonation, and light irradiation alone, nor is it evidence of a novel killing pathway.

### 3.5. In Vitro Cytocompatibility and Hemocompatibility of Nanomaterials

**Figure 5:**
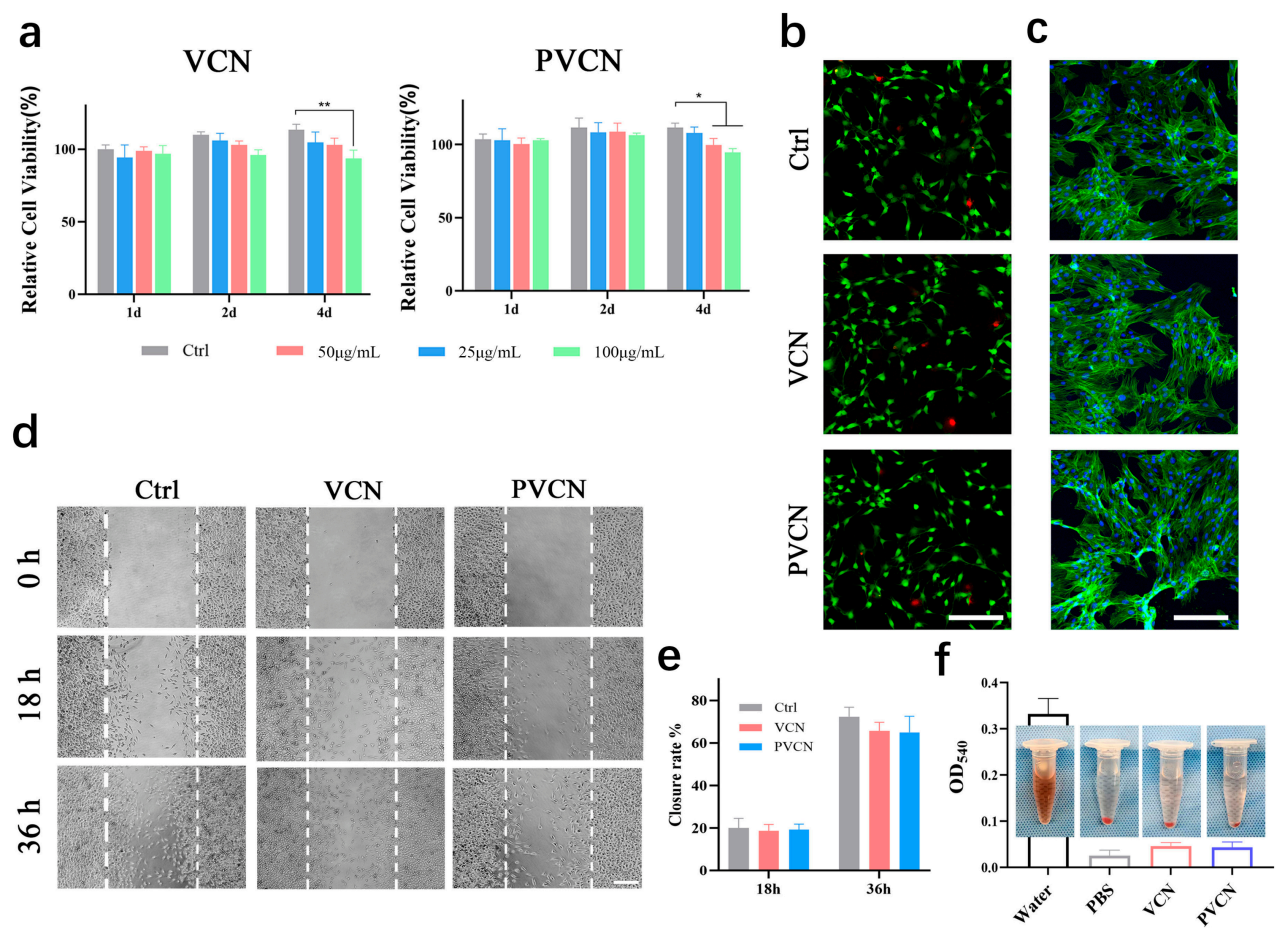
In vitro biocompatibility of the nanomaterials. (**a**) CCK-8 assay of rBMSCs cultured with VCN or PVCN. (**b**) Representative live/dead staining images of rBMSCs after 1 day of incubation with the nanomaterials, with scale bars of 500 µm. (**c**) Fluorescence images showing rBMSCs morphology after 1 day of incubation with the nanomaterials, with scale bars of 100 µm. (**d**) Representative wound-healing images of L929 fibroblasts after scratch treatment in the presence of the nanomaterials. (**e**) Quantitative analysis of wound closure rates. (**f**) Hemolysis assay results, including absorbance values and representative photographs of rat blood cell suspensions. Data are presented as mean ± SD (n = 3). * *p* < 0.05, ** *p* < 0.01.

Biocompatibility is an important factor for determining whether antibacterial nanomaterials can be further explored for biomedical applications. Therefore, rat bone marrow stem cells (rBMSCs) were used to evaluate the cytotoxicity of the nanomaterials using a CCK-8 assay and microscopic observation of morphology. As illustrated in Figure 5a, even at a high concentration of 200 µg mL^−1^, nanomaterials did not exhibit significant cell growth inhibition after co-culturing with cells for 1 and 2 days. After incubation for 4 days, rBMSCs maintained ideal viability at 50 and 100 µg mL^−1^; however, the cell activity was affected to some extent when nanomaterials were applied at 200 µg mL^−1^. Consistent results were obtained from live/dead fluorescence staining (Figure 5b). In addition, we used phalloidin/DAPI staining to observe the morphology of the co-cultured cells. As shown in Figure 5c, cells in all groups spread well, showing a polygonal shape with a number of filopodia. These results demonstrated that the nanomaterial exhibited favorable general cytocompatibility for rBMSCs within the tested concentration range.

In addition, a scratch wound healing assay was performed using L929 fibroblasts to evaluate the cytocompatibility of the material. The cell migration in the treated groups was comparable to that in the control group, with no significant inhibition (Figure 5d,e). These findings indicated that the material did not adversely affect fibroblast migratory capacity. Given the essential role of fibroblast migration in periodontal tissue repair, the scratch assay results further support the favorable in vitro biocompatibility of PVCN.

A hemolysis assay was conducted to evaluate the blood compatibility of the material. The absorbance values of the treated samples were comparable to those of the negative control and markedly lower than those of the positive control (Figure 5f), indicating minimal hemolytic activity. These findings suggested that the nanomaterial caused negligible erythrocyte damage and possessed favorable hemocompatibility.

### 3.6. In Vivo Evaluation in a Mouse Periodontitis Model

**Figure 6:**
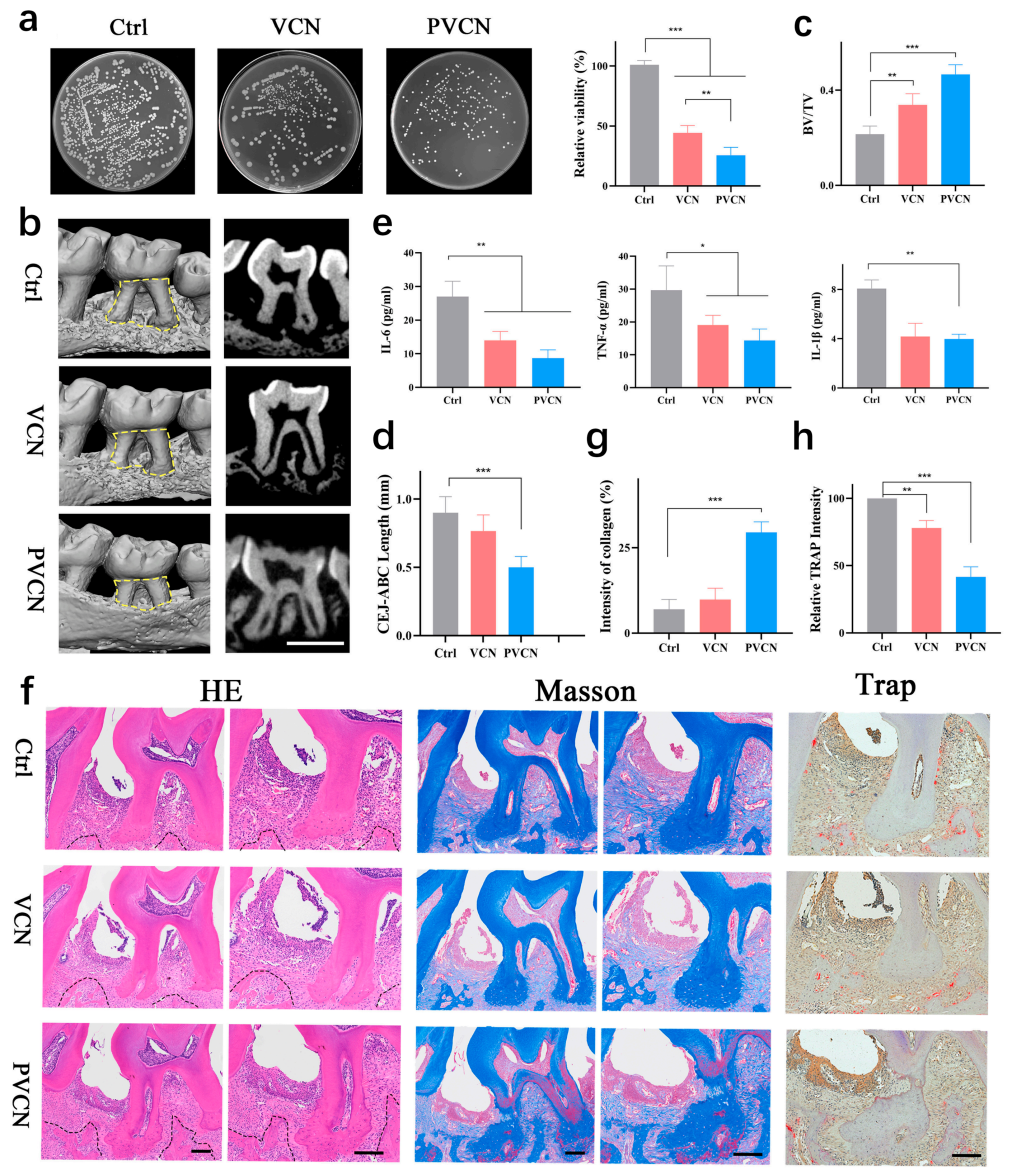
In vivo performance of the nanomaterials in the mouse model of periodontitis. (**a**) Representative bacterial colony images recovered from the tooth surfaces of mice and the corresponding quantitative analysis of bacterial viability. (**b**) Representative 3D reconstructed micro-CT images (left) and corresponding cross-sectional views (right) of alveolar bone in each group. Yellow dashed lines indicate the region of alveolar bone loss used for bone-volume analysis, with scale bar of 1 mm. (**c**) Quantification of BV/TV. (**d**) Quantification of the CEJ-ABC distance. (**e**) Serum levels of IL-6, TNF-α, and IL-1β in each group. (**f**) Representative histological images of periodontal tissues stained with H&E, Masson’s trichrome, and TRAP. The dashed area represents the alveolar bone, with scale bars of 200 µm. (**g**) Quantification of collagen staining intensity in each group. (**h**) Quantification of TRAP staining intensity in each group. Data are presented as mean ± SD (n = 5). * *p* < 0.05, ** *p* < 0.01, *** *p* < 0.001.

Based on their promising in vitro antibacterial performance and preliminary biocompatibility profile, a ligature-induced experimental periodontitis model was established in mice using silk ligatures to promote plaque accumulation [43], thereby evaluating the potential periodontal application of PVCN in vivo. First, CFU counting was used to quantitatively analyze residual bacterial levels. Compared with the control and VCN groups, the number of bacterial colonies in the PVCN group was significantly reduced, indicating a strong PDT effect in vivo (Figure 6a).

Micro-computed tomography (micro-CT) was performed to comprehensively evaluate the alveolar bone destruction in an experimental periodontitis model (Figure 6b). Three-dimensional reconstructed images revealed pronounced alveolar bone resorption in the untreated group, and markedly preserved alveolar bone architecture and reduced resorptive defects in the nanomaterial-treated group. Two-dimensional cross-sectional analyses revealed improved trabecular organization and high bone density in the interradicular region between the mesial and distal roots after treatment. Moreover, bone morphometric assessment revealed a significant increase in the bone volume fraction (BV/TV) within the region of interest compared with that in the control group (Figure 6c). Consistently, quantitative measurement of the distance between the cemento-enamel junction (CEJ) and alveolar bone crest (ABC) at the furcation of the first molar demonstrated a significant decrease in the treated groups (Figure 6d), indicating attenuation of bone loss. Collectively, these findings indicated that PVCN effectively alleviated alveolar bone resorption, likely by suppressing plaque biofilm-induced inflammatory responses, thereby contributing to periodontal tissue preservation in vivo. In addition, the treatment groups exhibited significantly lower serum levels of interleukin (IL)-6, IL-1β, and tumor necrosis factor-α (TNF-α) than the control group (Figure 6e), indicating alleviation of inflammation associated with periodontal tissue destruction [44].

Histological staining further revealed periodontal tissue changes in vivo (Figure 6f). In the control group, H&E staining showed obvious widening of the periodontal ligament and severe bone loss at the molar furcation area. Masson’s trichrome staining revealed disorganized and reduced collagen fibers in the periodontal ligament. TRAP staining revealed numerous TRAP-positive osteoclasts along the surface of alveolar bone, indicating active bone resorption. In contrast, the treated groups exhibited more organized collagen deposition (Figure 6g) and fewer osteoclasts (Figure 6h), suggesting suppressed bone destruction and improved periodontal tissue condition. Collectively, PVCN showed inflammatory periodontal reduction in the mouse ligature-induced model, likely through a PDT-mediated reduction in plaque-associated bacterial burden and the subsequent attenuation of inflammatory bone resorption.

The in vivo biocompatibility of the nanomaterials was further assessed through blood routine analysis and histopathological evaluation of major organs. H&E staining revealed no noticeable structural abnormalities or tissue injury in vital organs across all groups (Figure S4). Collectively, these findings support the favorable in vivo safety profile under the tested conditions of the nanomaterials.

## 4. Discussion

In this study, defect engineering and protonation were integrated to construct a protonated defect-engineered g-C_3_N_4_ nano-system (PVCN) with improved photodynamic antibacterial performance. Our results suggest that these two modifications play different but complementary roles. Defect engineering mainly improved the light-response characteristics of g-C_3_N_4_, as reflected by the broadened visible light absorption, narrowed band gap, and reduced photoluminescence intensity, whereas protonation primarily altered the surface physicochemical properties of the material, including its surface charge and interlayer stacking state [26,45]. This interpretation is generally consistent with previous studies showing that structural and electronic regulation is an effective way to improve the performance of g-C_3_N_4_-based materials, while protonation can further change layer organization and surface behavior without necessarily destroying the basic conjugated framework [27].

Importantly, the enhanced antibacterial performance of PVCN cannot be explained simply by stronger photodynamic activity. A key finding of the present study is that protonation also strengthened bacteria–material interaction. This result is meaningful because ROS generated during PDT are short-lived and act over a limited distance, so the efficiency of bacterial killing depends not only on how many ROS are produced, but also on whether they are generated sufficiently close to the bacterial surface. Previous work has likewise suggested that surface charge modulation and cell-surface attachment can substantially influence photocatalytic oxidative disinfection [39]. However, the present results do not fully decouple the contribution of protonation-mediated surface charge/adhesion from the enhancement of ROS generation, and the improved antibacterial efficacy of PVCN should therefore be interpreted as the combined outcome of strengthened bacteria–material interaction and enhanced photodynamic activity.

The downstream bacterial responses observed in this study further support this interpretation. SEM and TEM showed marked structural damage after treatment, while ATP depletion and extracellular protein leakage indicated membrane disruption and loss of cellular integrity. At the transcriptional level, genes and pathways related to membrane and cell wall organization, transmembrane transport, oxidative stress response, ion homeostasis, and DNA damage repair were significantly altered. These omics data should be interpreted in two layers. Oxidative stress-, membrane/wall organization-, and transport-related changes are consistent with the directly observed ROS accumulation and envelope damage, whereas changes in redox metabolism, DNA repair, energy metabolism, and translation likely represent secondary adaptive responses to PDT-induced stress. These findings provide mechanistic confirmation that PVCN induces classical ROS-mediated bacterial injury. Compared with previous studies that mainly inferred interfacial effects from surface potential changes or antibacterial outcomes [25,39], the present work provides a more complete line of evidence by combining AFM-based force measurement with intracellular ROS analysis and transcriptomic profiling. Therefore, we confirmed that the improved antibacterial performance of PVCN arises not from a new killing mechanism, but from the combination of enhanced photodynamic activity and protonation-strengthened bacterial adsorption.

Our results also help place protonation in a more specific antibacterial context. Recent work on protonated carbon nitride has shown that protonation can improve photocatalytic sterilization efficiency, partly through enhanced oxidative damage and increased physical disruption of bacterial cells [25]. The present study is in line with that general conclusion, but further suggests that the contribution of protonation here is not only physicochemical, but also bio-interfacial. This distinction is important because many earlier g-C_3_N_4_-related studies emphasized activity optimization itself, whereas our data suggest that interface regulation should be considered an equally important design variable.

This bio-interface-oriented interpretation is particularly relevant in the context of periodontitis. Periodontal infection develops in a biofilm-rich and spatially confined microenvironment, where local retention and effective contact of antibacterial agents can strongly influence treatment outcome [37]. Recent dental PDT studies have also noted that, despite promising in vitro activity, therapeutic efficacy in oral applications may be limited by insufficient retention of the photosensitizer at the target site [46]. In our study, PVCN showed strong activity against planktonic bacteria and biofilms in vitro. Although this simplified model does not fully reproduce the complexity of multispecies periodontal biofilms, it provides supportive evidence for the biofilm-disruptive capacity of PVCN and supports its potential application in local antibacterial therapy.

Although tissue samples, ex vivo models, or well-controlled in vitro systems may partially reproduce some oral conditions, such as tissue contact, saliva or crevicular fluid interference, biofilm presence, and local material retention, they cannot fully simulate disease progression, immune-inflammatory regulation, bone remodeling, or systemic biosafety after repeated treatment. Therefore, the ligature-induced mouse periodontitis model was used to evaluate the in vivo therapeutic feasibility of PVCN-mediated PDT.

From a translational perspective, the present in vivo protocol should be regarded as a proof-of-concept rather than a directly optimized clinical regimen. Human periodontal pockets present additional barriers, including deeper and narrower subgingival anatomy, saliva and crevicular fluid flow, bleeding, residual calculus or biofilm, and limited light penetration. Therefore, before clinical translation can be proposed, the following issues should be considered and addressed in future studies. Regarding the delivery strategy, attention should be paid to improving the retention of the material within periodontal pockets and minimizing interference from saliva and blood. Subgingival delivery systems, or combination with mucoadhesive agents or gel-based formulations, may be considered. Formulation strategies such as mucoadhesive gels or controlled local delivery systems may help reduce the required dose by improving retention at the target site, while minimizing potential cytotoxicity. Regarding the irradiation strategy, optical fibers or periodontal irradiation tips could be used to deliver light into deep periodontal pockets. The optimal irradiation time and intensity should be further explored by carefully balancing therapeutic efficacy and biosafety. In addition, PVCN-based PDT may be integrated with conventional periodontal procedures, such as scaling and root planing, to achieve more efficient periodontal treatment. These strategies would help address the concerns regarding local retention, long-term stability, light accessibility, and repeated-use safety of PVCN in periodontal environments.

Several limitations should nevertheless be acknowledged. First, *S. aureus* and *E. coli* were used as representative bacteria for preliminary broad-spectrum antibacterial screening, and a mono-species *S. aureus* biofilm was used for initial anti-biofilm evaluation. These models are useful for assessing general light-activated antibacterial activity, but they do not fully reproduce the dysbiotic, anaerobic, polymicrobial, and matrix-rich nature of subgingival periodontal biofilms. Therefore, the present in vitro findings should not be directly extrapolated to validated efficacy against periodontal pathogens or clinically representative periodontal biofilms. Future studies should include periodontally relevant oral species, such as *F. nucleatum*, *A. actinomycetemcomitans*, *Actinomyces* spp., and preferably saliva- or plaque-derived multispecies biofilm models, to further validate the periodontal applicability of PVCN. Second, the mechanistic experiments did not include a complete set of parallel dark/light and VCN/PVCN controls. In particular, transcriptomic analysis was performed by comparing untreated S. aureus with PVCN plus visible light irradiation, without additional groups exposed to light alone, PVCN in the dark, or VCN plus irradiation. Therefore, the transcriptomic changes should be interpreted as downstream bacterial responses to PVCN-mediated PDT treatment, rather than definitive evidence that all observed responses are specifically attributable to protonation-mediated adhesion. Moreover, the contribution of surface charge-mediated adhesion was not fully decoupled from the enhanced ROS generation. Future studies using ROS scavengers, selected marker validation, comparative VCN/PVCN dark/light controls, and charge-controlled or charge-neutralized material systems would help more precisely distinguish the roles of electrostatic bacteria–material interaction and ROS production in the antibacterial process. Third, the cytocompatibility assays using rBMSCs and L929 fibroblasts provide preliminary general biocompatibility information rather than definitive periodontal biosafety. Because they do not fully represent the periodontal microenvironment, additional evaluation using human gingival fibroblasts, periodontal ligament cells, oral epithelial cells, and more clinically relevant exposure conditions is required. Fourth, the in vivo experiment was conducted over a relatively short treatment period and, as mentioned above, was performed only in a mouse experimental periodontitis model. The long-term therapeutic outcomes, local retention behavior, degradation or clearance, and repeated-use safety of PVCN in periodontal environments remain to be systematically evaluated. In clinically relevant periodontal pockets, the effective local concentration and exposure duration of PVCN may be affected by saliva, blood, gingival crevicular fluid flow, residual biofilm, and material clearance. In addition, repeated administration may lead to potential material accumulation, local inflammatory responses, or tissue irritation, which requires long-term safety evaluation. Therefore, future studies should further optimize the local dose, exposure duration, treatment frequency, and delivery formulation to establish a more precise and clinically relevant therapeutic window for periodontal application.

Overall, the present study supports a design concept that goes beyond conventional photoactivity optimization alone. By combining defect engineering with protonation, PVCN achieves both improved photodynamic performance and strengthened bacterial adsorption, and these two effects appear to work together to determine antibacterial efficacy. From this perspective, our work supports protonated defect-engineered g-C_3_N_4_ as a promising bio-interface-enhanced PDT material. Periodontitis should be considered a potential application scenario supported by preliminary in vitro and in vivo proof-of-concept data. However, further validation using periodontally relevant pathogens, clinically representative multispecies biofilms, and optimized periodontal delivery and irradiation strategies is required before PVCN can be considered a disease-specific periodontal therapeutic system.

## 5. Conclusions

In this study, we engineered a protonated defect-modified carbon nitride material with a stable positively charged surface, PVCN, that enhances the visible light photodynamic activity. The strengthened electrostatic interactions between PVCN and negatively charged bacterial membranes markedly improved interfacial contact and facilitated efficient ROS diffusion, thereby establishing a highly effective interface-enhanced PDT mode. Under mild visible light irradiation, PVCN efficiently killed planktonic bacteria and disrupted mono-species *S. aureus* biofilms, while showing favorable preliminary cytocompatibility and hemocompatibility. Omics analyses further revealed that the superior bacterial adhesion capacity of PVCN amplifies ROS-mediated disruption of membrane integrity and metabolic pathways, ultimately leading to bacterial death. In a ligature-induced periodontitis model, PVCN reduced bacterial burden, inflammatory cytokine levels, osteoclast-mediated bone resorption, and alveolar bone loss. Collectively, these findings demonstrate the antibacterial potential and in vivo therapeutic promise of PVCN in an experimental periodontitis model. Nevertheless, further in vitro studies against periodontal pathogens and more clinically representative in vivo investigations remain necessary before its efficacy against periodontitis can be fully established.

## Data Availability

The original contributions presented in this study are included in the article/Supplementary Materials. Further inquiries can be directed to the corresponding author.

## Supplementary Materials

The following supporting information can be downloaded at: https://www.mdpi.com/article/10.3390/ma19112191/s1, Figure S1: Antibacterial effects of nanomaterials in different concentration and under irradiation for different time; Figure S2: Disruption of bacterial membrane integrity induced by nanomaterial treatment; Figure S3: The representative interaction force profile between nanomaterials and bacteria cells examined by AFM; Figure S4: H&E staining of major organs (heart, liver, spleen, lung, and kidney).

## Abbreviations

The following abbreviations are used in this manuscript:

ABC	alveolar bone crest
AFM	atomic force microscopy
ANOVA	analysis of variance
ATCC	American Type Culture Collection
ATP	adenosine triphosphate
BV/TV	bone volume/total volume
CCK-8	Cell Counting Kit-8
CEJ	cemento-enamel junction
CFU	colony-forming units
CL	control group
COG	Clusters of Orthologous Groups
DEGs	differentially expressed genes
DMEM	Dulbecco’s modified Eagle’s medium
*E. coli*	*Escherichia coli*
ESR	electron spin resonance
FBS	fetal bovine serum
FTIR	Fourier-transform infrared spectroscopy
GO	Gene Ontology
g-C_3_N_4_	graphitic carbon nitride
H&E	hematoxylin and eosin
IL	interleukin
KEGG	Kyoto Encyclopedia of Genes and Genomes
LB	Luria–Bertani
LSCM	laser scanning confocal microscopy
OD	optical density
PBS	phosphate-buffered saline
PCA	principal component analysis
PDT	photodynamic antibacterial therapy
PL	photoluminescence
PVCN	protonated defect-engineered atomic-layered graphitic carbon nitride nano-system
rBMSCs	rat bone marrow–derived mesenchymal stem cells
RhB	rhodamine B
ROS	reactive oxygen species
*S. aureus*	*Staphylococcus aureus*
SD	standard deviation
SEM	scanning electron microscopy
TEM	transmission electron microscopy
TNF-α	tumor necrosis factor-α
TR	treatment group
TRAP	tartrate-resistant acid phosphatase
XPS	X-ray photoelectron spectroscopy
XRD	X-ray diffraction

## References

[1] Mainas G., Ide M., Rizzo M., Magan-Fernandez A., Mesa F., Nibali L. (2022). Managing the Systemic Impact of Periodontitis. Medicina.

[2] Hashim N.T., Babiker R., Padmanabhan V., Ahmed A.T., Chaitanya N.C.S.K., Mohammed R., Priya S.P., Ahmed A., El Bahra S., Islam M.S. (2025). The Global Burden of Periodontal Disease: A Narrative Review on Unveiling Socioeconomic and Health Challenges. Int. J. Environ. Res. Public Health.

[3] Kinane D.F., Stathopoulou P.G., Papapanou P.N. (2017). Periodontal diseases. Nat. Rev. Dis. Primers.

[4] Sanz M., Herrera D., Kebschull M., Chapple I., Jepsen S., Berglundh T., Sculean A., Tonetti M.S., Participants E.F.P.W., Methodological C. (2020). Treatment of stage I–III periodontitis—The EFP S3 level clinical practice guideline. J. Clin. Periodontol..

[5] Ho C.S., Wong C.T.H., Aung T.T., Lakshminarayanan R., Mehta J.S., Rauz S., McNally A., Kintses B., Peacock S.J., de la Fuente-Nunez C. (2025). Antimicrobial resistance: A concise update. Lancet Microbe.

[6] Nguyen V.-N., Zhao Z., Tang B.Z., Yoon J. (2022). Organic photosensitizers for antimicrobial phototherapy. Chem. Soc. Rev..

[7] Songca S.P., Adjei Y. (2022). Applications of Antimicrobial Photodynamic Therapy against Bacterial Biofilms. Int. J. Mol. Sci..

[8] Youf R., Müller M., Balasini A., Thétiot F., Müller M., Hascoët A., Jonas U., Schönherr H., Lemercier G., Montier T. (2021). Antimicrobial Photodynamic Therapy: Latest Developments with a Focus on Combinatory Strategies. Pharmaceutics.

[9] Surur A.K., de Oliveira A.B., De Annunzio S.R., Ferrisse T.M., Fontana C.R. (2024). Bacterial resistance to antimicrobial photodynamic therapy: A critical update. J. Photochem. Photobiol. B Biol..

[10] Cheng H., Wang N., Yang Y., Jiao X., Han P., Duan W., Huang D., An M., Chen W., Yao X. (2023). The enhanced visible light driven photocatalytic activity of zinc porphyrin/g-C3N4 nanosheet for efficient bacterial infected wound healing. J. Colloid Interface Sci..

[11] Jia R., He C., Wang S., Gao Y., Song L., Wang P., Liao G., Shi X. (2024). Recent advances in graphitic carbon nitride-based heterojunction for biomedical applications. Chem. Eng. J..

[12] Chen L., Maigbay M.A., Li M., Qiu X. (2024). Synthesis and modification strategies of g-C3N4 nanosheets for photocatalytic applications. Adv. Powder Mater..

[13] Stefa S., Zografaki M., Dimitropoulos M., Paterakis G., Galiotis C., Sangeetha P., Kiriakidis G., Konsolakis M., Binas V. (2023). High surface area g-C3N4 nanosheets as superior solar-light photocatalyst for the degradation of parabens. Appl. Phys. A.

[14] Shahbaz A., Afaf A., Tahir N., Ullah A., Saim S. (2021). Non Precious Metal Catalysts: A Fuel Cell and ORR Study of Thermally Synthesized Nickel and Platinum Mixed Nickel Nanotubes for PEMFC. Key Eng. Mater..

[15] Guo Y., Liu G., Yin W., Zhang Y., Shi L. (2024). Precise defect engineering g-C3N4 fabrication to improve hydrogen production performance. Fuel.

[16] Hou S., Gao X., Lv X., Zhao Y., Yin X., Liu Y., Fang J., Yu X., Ma X., Ma T. (2024). Decade Milestone Advancement of Defect-Engineered g-C3N4 for Solar Catalytic Applications. Nano-Micro Lett..

[17] Ba G., Hu H., Bi F., Yu J., Liu E., Ye J., Wang D. (2025). Engineering nitrogen vacancies and cyano groups into C3N4 nanosheets for highly efficient photocatalytic H2O2 production. Appl. Catal. B Environ. Energy.

[18] Feng C., Tang L., Deng Y., Wang J., Liu Y., Ouyang X., Yang H., Yu J., Wang J. (2021). A novel sulfur-assisted annealing method of g-C3N4 nanosheet compensates for the loss of light absorption with further promoted charge transfer for photocatalytic production of H2 and H2O2. Appl. Catal. B Environ..

[19] Hamblin M.R., Hasan T. (2004). Photodynamic therapy: A new antimicrobial approach to infectious disease?. Photochem. Photobiol. Sci..

[20] Karagulyan M., Goebel M.-O., Diehl D., Abu Quba Abd A., Kästner M., Bachmann J., Wick Lukas Y., Schaumann Gabriele E., Miltner A. (2022). Water Stress-Driven Changes in Bacterial Cell Surface Properties. Appl. Environ. Microbiol..

[21] Hoffmann T.D., Reeksting B.J., Gebhard S. (2021). Bacteria-induced mineral precipitation: A mechanistic review. Microbiology.

[22] Hang N.T., Zhang S., Yang W. (2017). Efficient exfoliation of g-C3N4 and NO2 sensing behavior of graphene/g-C3N4 nanocomposite. Sens. Actuators B Chem..

[23] Subbiah M., Mariappan A., Sundaramurthy A., Venkatachalam S., Renganathan R.T., Saravanan N., Pitchaimuthu S., Srinivasan N. (2024). Protonated C3N4 Nanosheets for Enhanced Energy Storage in Symmetric Supercapacitors through Hydrochloric Acid Treatment. ACS Omega.

[24] Liao G., He F., Li Q., Zhong L., Zhao R., Che H., Gao H., Fang B. (2020). Emerging graphitic carbon nitride-based materials for biomedical applications. Prog. Mater. Sci..

[25] Zhu X., Xu C., Mao J., Zhang Y., Bai Y. (2025). Protonated carbon nitride for rapid photocatalytic sterilization via synergistic oxidative damage and physical destruction. J. Environ. Sci..

[26] Ong W.-J., Tan L.-L., Chai S.-P., Yong S.-T., Mohamed A.R. (2015). Surface charge modification via protonation of graphitic carbon nitride (g-C3N4) for electrostatic self-assembly construction of 2D/2D reduced graphene oxide (rGO)/g-C3N4 nanostructures toward enhanced photocatalytic reduction of carbon dioxide to methane. Nano Energy.

[27] Kang H.-J., Lee T.-G., Bari G.A.K.M.R., Seo H.-W., Park J.-W., Hwang H.J., An B.-H., Suzuki N., Fujishima A., Kim J.-H. (2021). Sulfuric Acid Treated g-CN as a Precursor to Generate High-Efficient g-CN for Hydrogen Evolution from Water under Visible Light Irradiation. Catalysts.

[28] Wang H., Li M., Li H., Lu Q., Zhang Y., Yao S. (2019). Porous graphitic carbon nitride with controllable nitrogen vacancies: As promising catalyst for enhanced degradation of pollutant under visible light. Mater. Des..

[29] Song S., Zou X., Kang Y., Ji X., Lu R., Yu A. (2023). Single-Color and Two-Color Femtosecond Pump–Probe Experiments on Graphitic Carbon Nitrides Revealing Their Charge Carrier Kinetics. J. Phys. Chem. C.

[30] Shang Y., Wang Y., Lv C., Jing F., Liu T., Li W., Liu S., Chen G. (2022). A broom-like tube-in-tube bundle O-doped graphitic carbon nitride nanoreactor that promotes photocatalytic hydrogen evolution. Chem. Eng. J..

[31] Zhang W., Dong M., Jiang K., Yang D., Tan X., Zhai S., Feng R., Chen N., King G., Zhang H. (2022). Self-repairing interphase reconstructed in each cycle for highly reversible aqueous zinc batteries. Nat. Commun..

[32] Liu Y., Chen X., Kamali M., Rossi B., Appels L., Dewil R. (2024). Unraveling the Presence and Positions of Nitrogen Defects in Defective g-C3N4 for Improved Organic Photocatalytic Degradation: Insights from Experiments and Theoretical Calculations. Adv. Funct. Mater..

[33] Niu P., Yin L.-C., Yang Y.-Q., Liu G., Cheng H.-M. (2014). Increasing the Visible Light Absorption of Graphitic Carbon Nitride (Melon) Photocatalysts by Homogeneous Self-Modification with Nitrogen Vacancies. Adv. Mater..

[34] Saxena D., Maitra R., Bormon R., Czekanska M., Meiers J., Titz A., Verma S., Chopra S. (2023). Tackling the outer membrane: Facilitating compound entry into Gram-negative bacterial pathogens. npj Antimicrob. Resist..

[35] Jiao C., Gong S., Shi M., Guo L., Jiang Y., Man C. (2023). Depletion of reactive oxygen species induced by beetroot (Beta vulgaris) extract leads to apoptosis-like death in Cronobacter sakazakii. J. Dairy Sci..

[36] Zhu S., Chen Y., Yang M., Lin X., Fang Y., Liu L., Kang J. (2026). Antibacterial mechanism of action of perilla leaf essential oil on Shigella flexneri and its preservation effectiveness in fresh-cut lettuce. Int. J. Food Microbiol..

[37] Papapanou P.N., Sanz M., Buduneli N., Dietrich T., Feres M., Fine D.H., Flemmig T.F., Garcia R., Giannobile W.V., Graziani F. (2018). Periodontitis: Consensus report of workgroup 2 of the 2017 World Workshop on the Classification of Periodontal and Peri-Implant Diseases and Conditions. J. Clin. Periodontol..

[38] Zhu G.-Y., Lu B.-Y., Zhang T.-X., Zhang T., Zhang C.-L., Li Y., Peng Q. (2018). Antibiofilm Effect of Drug-Free and Cationic Poly(D,L-Lactide-Co-Glycolide) Nanoparticles Via Nano–Bacteria Interactions. Nanomedicine.

[39] Zhu Z., Bao L., Pestov D., Xu P., Wang W.-N. (2023). Cellular-level insight into biointerface: From surface charge modulation to boosted photocatalytic oxidative disinfection. Chem. Eng. J..

[40] Chen Y., Xue Y., Xu X., Su Y., Tong X., Zhu L., Zuo Y., Ban C., Chen J., Que W. (2025). Tetrazine-enhanced donor-acceptor-donor metal-organic frameworks for photodynamic antibacterial therapy and wound healing. Nat. Commun..

[41] Zhong C., He Y., Zou J., Gao L., Wang J., Zhu J., Xue W., Gou S., Zhang Y., Liu H. (2025). An antimicrobial peptide as a potential therapy for bacterial pneumonia that alleviates antimicrobial resistance. Nat. Commun..

[42] Song G., Zhang S., Tian M., Zhang L., Guo R., Zhuo W., Yang M. (2021). Molecular insights into the human ABCB6 transporter. Cell Discov..

[43] Hiyari S., Wong R.L., Yaghsezian A., Naghibi A., Tetradis S., Camargo P.M., Pirih F.Q. (2018). Ligature-induced peri-implantitis and periodontitis in mice. J. Clin. Periodontol..

[44] Hajishengallis G., Chavakis T. (2021). Local and systemic mechanisms linking periodontal disease and inflammatory comorbidities. Nat. Rev. Immunol..

[45] Zhang Y., Sun S., Wu Y., Chen F. (2024). Emerging Roles of Graphitic Carbon Nitride-based Materials in Biomedical Applications. ACS Biomater. Sci. Eng..

[46] Monteiro C.M., Gonçalves J., Machado G.B., Chiarelli-Neto O., Prates R.A., Frochot C., Pavani C. (2025). Viscous methylene blue formulation for antimicrobial photodynamic therapy in dentistry. Sci. Rep..

