# Supplementary material for: Protonated Defect-Engineered Carbon Nitride Enables Bio-Interface-Enhanced Photodynamic Antibacterial Activity with Potential Periodontal Application
Source: Materials (Basel). 2026 May 22;19(11):2191. doi: 10.3390/ma19112191 (PMC13258522; doi:10.3390/ma19112191)
Supplement: Supplementary file 1 [file materials-19-02191-s001.zip › materials-4291602-supplementary.pdf]

Supplementary Materials

Protonated Defect-Engineered Carbon Nitride Enables Bio-Interface-Enhanced Photodynamic Antibacterial Activity with Potential Periodontal Application

Supplementary Figures

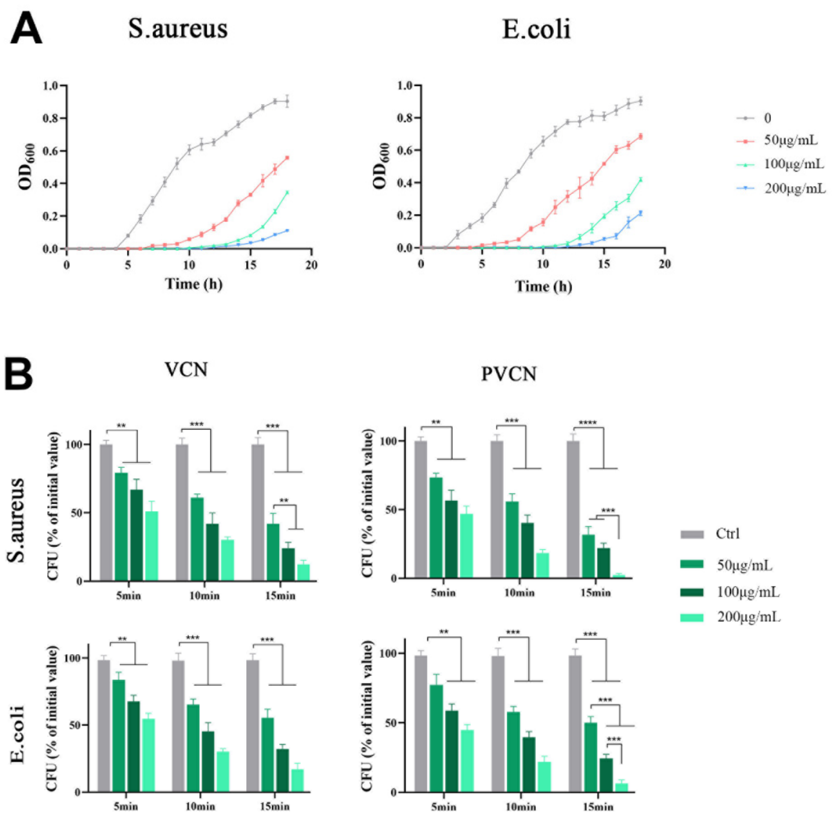

Figure S1. Antibacterial effects of nanomaterials in different concentration and under irradiation for different time. (**A**) The growth curve of bacteria. (**B**) The loss of viability of bacteria. The data are presented as mean ± SD (n = 3). Statistical significance: ** $p < 0.01$, *** $p < 0.001$ and **** $p < 0.0001$.

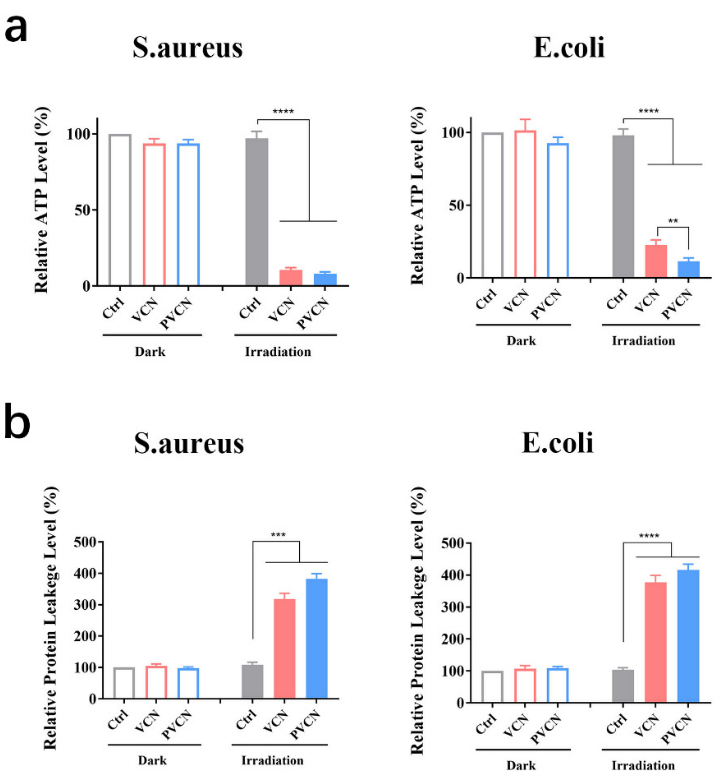

Figure S2. Disruption of bacterial membrane integrity induced by nanomaterial treatment. (**a**) The ATP level of bacteria after treatment with 200 μg mL^{-1} nanomaterials for 15 min. (**b**) Protein leakage of bacteria after treatment with 200 μg mL^{-1} nanomaterials for 15 min. The data are presented as mean ± SD (n = 3). Statistical significance: ** $p < 0.01$, *** $p < 0.001$ and **** $p < 0.0001$.

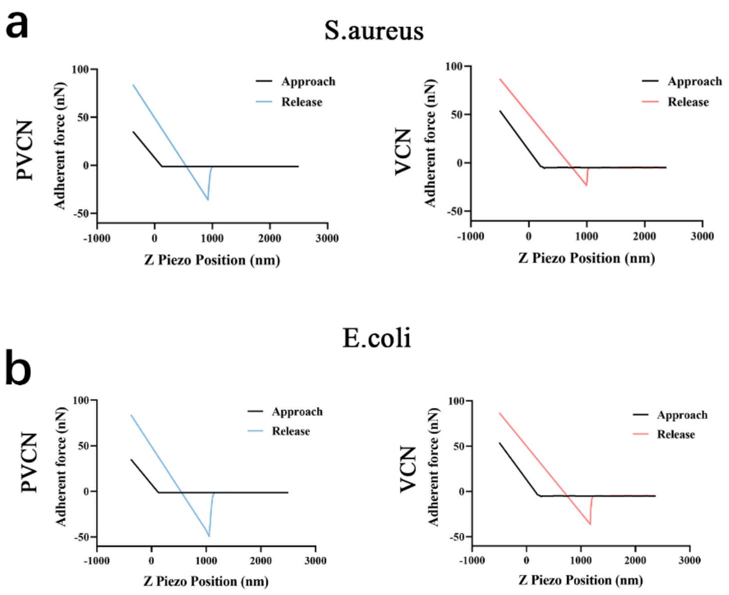

Figure S3. The representative interaction force profile between nanomaterials and bacteria cells examined by AFM. (**a**) Interaction force between nanomaterials and *S.aureus*. (**b**) Interaction force between nanomaterials and *E.coli*.

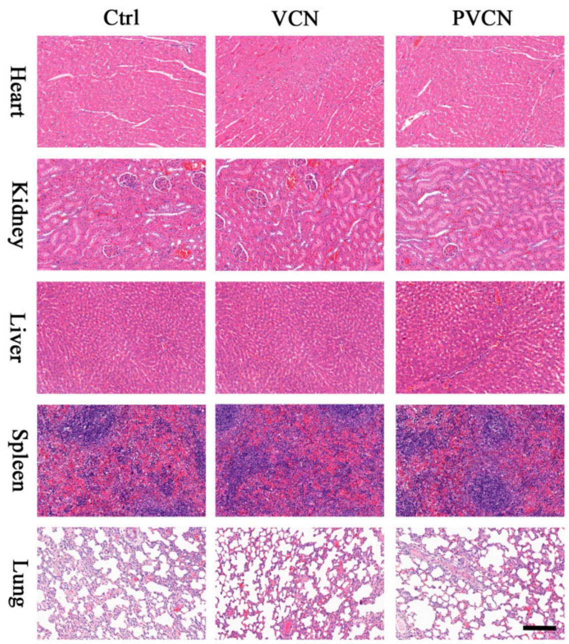

Figure S4. H&E staining of major organs (heart, liver, spleen, lung, and kidney). Scale bars are 200 μm.
